# Auditory and vibrotactile interactions in perception of timbre acoustic features

**DOI:** 10.1038/s41598-025-21908-4

**Published:** 2025-10-30

**Authors:** Loonan Chauvette, Anne Sophie Grenier, Philippe Albouy, Emily Coffey, Robert Zatorre, Andréanne Sharp

**Affiliations:** 1 CERVO Brain Research Centre Université Laval, G1E 1T2 Quebec City, Canada; 2 Faculty of Medicine, Université Laval, G1V 0A6 Quebec City, Canada; 3 School of Psychology, Laval University, Quebec City, G1V 0A6 Canada; 4 Concordia University, Montreal, H3G 1M8 Canada; 5 Montreal Neurological Institute McGill University, Montreal, H3A 2B4 Canada; 6 Centre for Research on Brain, Language and Music (CRBLM), McGill University, Montreal, H3G 2A8 Canada; 7 International Laboratory for Brain, Music and Sound Research (BRAMS), Montreal, H2V 2S9 Canada

**Keywords:** Sensory substitution, Multimodal perception, Vibrotactile, Timbre, Audition, Assistive technology, Auditory system, Somatosensory system, Perception

## Abstract

Recently, there has been increasing interest in developing auditory-to-vibrotactile sensory devices. However, the potential of these technologies is constrained by our limited understanding of which features of complex sounds can be perceived through vibrations. The present study aimed to investigate the vibrotactile perception of acoustic features related to timbre, an essential component to identify environmental, speech and musical sounds. Discrimination thresholds were measured for six features: three spectral (number of harmonics, harmonic roll-off ratio, even-harmonic attenuation) and three temporal (attack time, amplitude modulation depth and amplitude modulation frequency) using auditory, vibrotactile and combined auditory + vibrotactile stimulation in 31 adult humans with normal tactile and auditory sensitivity. Result revealed that all spectral and temporal features can be reliably discriminated via vibrotactile stimulation only. However, for spectral features, vibrotactile thresholds were significantly higher (i.e., worse) than auditory thresholds whereas, for temporal features, only vibrotactile amplitude modulation frequency was significantly higher. With simultaneous auditory and tactile presentation, thresholds significantly improved for attack time and amplitude modulation depth, but not for any of the spectral acoustic features. These results suggest that vibrotactile temporal cues have a more straightforward potential for assisting auditory perception, while vibrotactile spectral cues may require specialized signal processing schemes.

**Supplementary Information:**

The online version contains supplementary material available at 10.1038/s41598-025-21908-4.

## Introduction

Vibrotactile devices designed to convey sound through touch are seeing rapid development^1–3^. Despite the growing interest in auditory-to-vibrotactile sensory substitution devices (SSDs) across various fields such as rehabilitation, human-computer interaction, and music^4–6^, their adoption in real-world settings remains limited. This lack of widespread use does not seem to stem from a shortage of viable applications, which can range from assistive to entertainment purposes^7^, but rather from the numerous particularities and challenges of using touch as a sensory channel to transmit auditory information^8^. Indeed, some digital signal processing is often required to convey basic acoustic features (e.g., those involved in intensity and pitch perception) in a way that conforms to the tactile modality’s inherent perceptual abilities and limitations. However, transmitting more complex acoustical cues like spectral and temporal modulation features that produce the auditory perception of timbre – the character or quality of a sound as distinct from its pitch and intensity^9^ – remains challenging due to an incomplete understanding of how the tactile system can process these acoustic features. Designing effective auditory-to-vibrotactile SSDs thus requires further psychophysical investigation of human vibratory sensation^10^.

Efficient use of this sensory channel requires stimulating the adequate type of skin mechanoreceptors^11^. In particular, Pacinian corpuscles appear as an optimal target because of their high sensitivity and rapid adaptation to mechanical vibrations, allowing them to synchronize their firing to the cycle of periodic stimuli^12–14^. Although these mechanoreceptors share several psychophysical properties with hearing^15–17^, they exhibit some limitations, including a narrower frequency response range of 100 to 1000 Hz, which only partially overlaps with the broader 20 to 20,000 Hz range of hearing^17^. Additionally, their ability to discriminate between frequencies is lower, although this limitation appears to be trainable^18^. Some existing studies have provided valuable insights into absolute detection thresholds^1,19^, growth of perceived intensity^20,21^, and frequency discrimination^18,22^ for pure tones within the vibrotactile frequency range. However, few studies have investigated how the tactile modality processes complex signals with spectral or temporal modulations such as multiple harmonics or amplitude variations.

In hearing, spectro-temporal modulations are critical, as they underpin the perception of timbre^9^. Timbre is essential for recognizing speech sounds, identifying environmental cues, and distinguishing musical instruments^23–26^. It also plays a vital role in auditory scene analysis and speech perception in noisy environments^27–29^. The degradation of these modulations can severely impair auditory processing, especially temporal cues important for speech, and spectral cues important for melodic and harmonic perception^30,31^. This phenomenon is observed in cochlear implant users, who experience significant deficits in timbre perception, particularly in its spectral dimensions^32,33^. To substitute, correct, or augment hearing, auditory-to-vibrotactile SSDs must be able to convey these spectro-temporal modulations. However, our current understanding of complex vibrations perception remains incomplete^34–36^. Specifically, a key question is: which spectral and temporal features of complex tones can be reliably processed by the vibrotactile system, and which are beyond its sensory capabilities?

A further important question that we also address is whether the addition of vibrotactile input to an auditory task enhances discrimination thresholds, and if so for which features. Adding vibrotactile cues has previously been shown to improve low- and high-level perceptual processes such as detection thresholds^37,38^ or speech in noise comprehension^39,40^. However, it is also relevant to assess if there exist multimodal interactions in the perception of mid-level acoustic features that underlie fundamental aspects of auditory cognition, from sound identification to speech and music perception. The current research then focuses on the auditory, vibrotactile, and multimodal auditory + vibrotactile perception of six fundamental spectral and temporal features of complex signals. By comparing auditory, tactile, and bimodal discrimination abilities, this study seeks to inform the development of more effective auditory-to-vibrotactile SSDs and signal processing strategies capable of transmitting spectro-temporal modulations that are critical for timbre perception through vibrations, while also exploring the limitations of vibrotactile perception and its underlying mechanisms.

## Methods

### Participants

Thirty-one human adults (22 women, 9 men) aged from 20 to 48 years (M = 28.02, SD = 6.12) residing in Quebec City (Canada) participated in this study. Participants were recruited between June 2024 and October 2024 through university-wide mailing lists and social media. Ethics approval was granted from the Research Committee for Sectorial Research in Neuroscience and Mental Health of the CIUSSS—Capitale Nationale (Ethic approval number 2023–2695) and written informed consent was obtained from all participants. All methods were performed in accordance with the relevant guidelines and regulations.

Inclusion criteria were normal hearing, defined as hearing thresholds ≤ 25 dB HL from 0.25 to 8 kHz^41^, and no significant musical training, defined as ≤ 3 years of formal musical education outside of classes from the standard school curriculum. Exclusion criteria were (1) a diagnosis of a neurological, cognitive, or psychological disorder (e.g., autism spectrum disorder, attention deficit disorder); (2) a condition that could affect tactile perception (e.g., diabetes, Raynaud’s disease) or the skin of the hands (e.g., hyperhidrosis, eczema); and (3) uncorrected visual impairment (e.g., unable to clearly see the computer screen). Of the 47 initial volunteers, screening measures revealed that 15 did not meet the inclusion and exclusion criteria: three had diagnosed attention deficit disorder, two had hearing thresholds ≥ 25 dB HL, ten were found to have over three years of musical training upon completing the musical history questionnaire and were redirected to other ongoing studies in our lab involving musicians. One participant was also excluded after data collection for having a major outlier value in their vibrotactile threshold at 100 Hz based on Tukey’s fence method^42^. Socio-demographic characteristics of the final sample are presented in Table 1.

**Table 1:** Participant characteristics.

Characteristics	*n*	%
**Sex**		
Women	22	71.0
Men	9	29.0
**Handedness**		
Right	27	87.1
Left	4	12.9
**Highest diploma**		
College	6	19.4
Bac	7	22.6
Master	14	45.2
Doctorate	4	12.9

### Rationale for sample size

An a priori simulation-based power analysis for mixed-effects models^43^ was conducted using the R package *designr* (v0.1.13). One thousand simulated datasets were generated for various sample sizes using the mean random effect variance, mean residual variance, and smallest regression coefficient from a 12 participants pilot study^44^. For each sample size, power was calculated as the proportion of datasets yielding statistically significant results^45^. Results indicated that 25 participants were sufficient to achieve 80% power.

### Apparatus and materials

Socio-demographic information was collected through a custom questionnaire. Musical history was assessed using the French version of the Montreal Musical History Questionnaire (MMHQ), a 15-minute online questionnaire designed to help individuals recall and document their musical training experiences^46^. The hearing status of participants was confirmed with a screening procedure including external auditory canal visualization, tympanometry, and pure tone audiometry, conducted in a quiet room respecting the maximum permissible ambient noise level^47^ using an AA222 middle ear analyzer and audiometer (Interacoustics, Assens, Denmark).

**Fig. 1:**
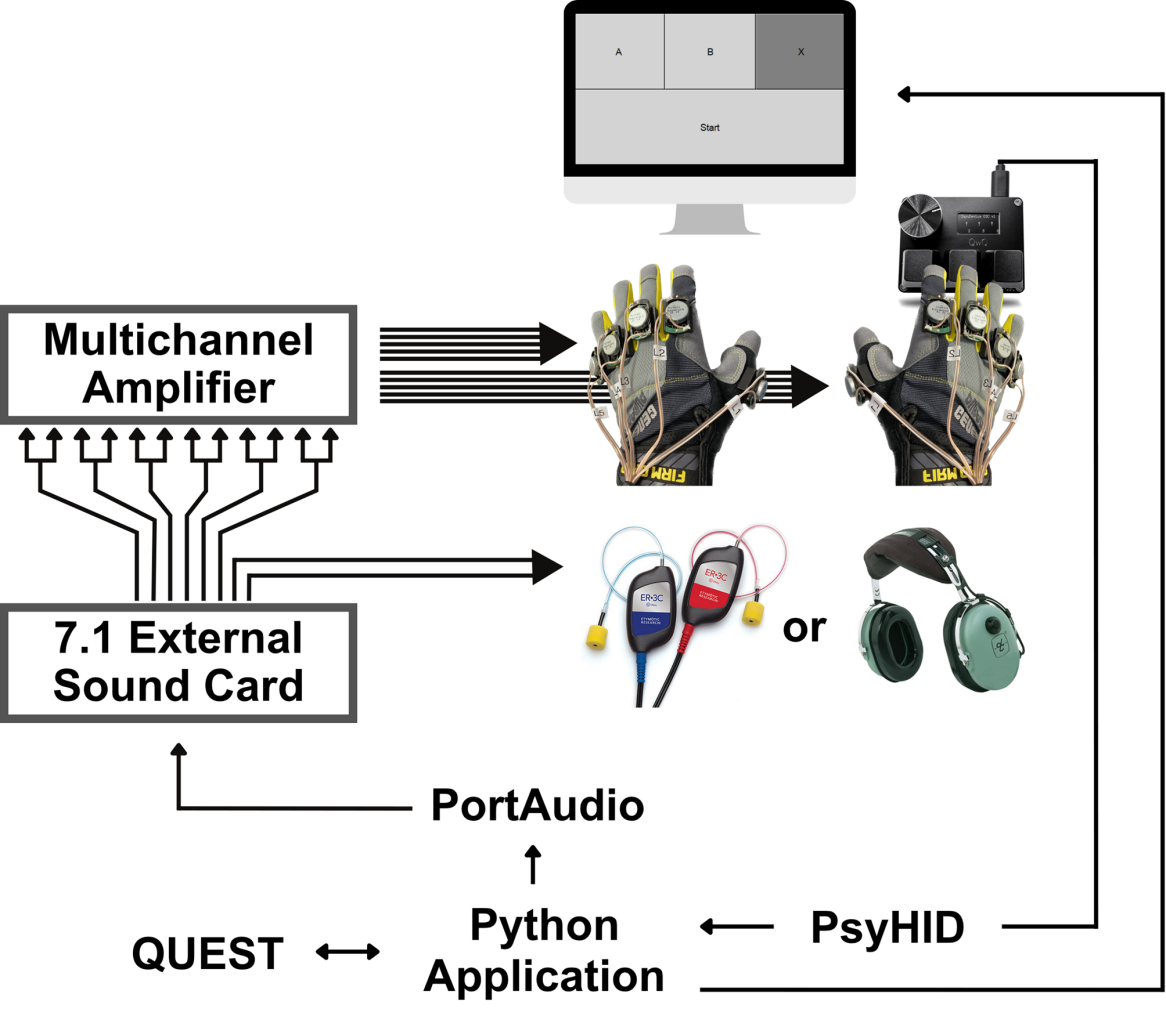
Experimental setup and apparatus used for the experiments.

Vibrotactile stimuli were delivered using the Multichannel Vibrotactile Gloves, which were validated in our previous methodological study^1^. In brief, this device enables the transmission of digital sounds as high-fidelity vibrotactile stimulations using 10 TEAX14C02-8 haptic audio exciters (Tectonic, New York, NY, USA) affixed to the back of each finger at the level of the proximal phalange on General Purpose leather gloves (Firm Grip, Atlanta, GA, USA) using custom 3D printed mounting pieces. The actuators powered by a 12-channel MA1260 Class D amplifier (DaytonAudio, Springboro, OH, USA) driven by an ICUSBAUDIO7D external sound card (StarTech.com Ltd., London, ON, Canada) with eight 16-bit digital to analog channels (Fig. 1). Auditory stimuli were presented through insert earphones (IP30, RadioEar, Middelfart, Denmark). For tactile only tasks, auditory masking was applied to prevent sounds emitted by the haptic actuators being heard through air or bone conduction. As in our previous studies^1,18^, participants wore earplugs and circumaural headphones (10 S/DC, David Clark, Worcester, MA, USA) playing white noise. To confirm sufficient masking and attenuation, five participants completed a two-interval forced-choice detection task without wearing the gloves for stimuli 10 dB higher than those used for the study, which showed a performance at chance level.

Responses were recorded using a three-button keyboard (SayoDevice 3 °C, Newhui, Rowland Heights, CA, USA). Stimuli were generated and presented using custom Python (Python Software Foundation) scripts on a Windows 11 laptop. Stimuli presentation and responses collection was achieved using PsychPortAudio for audio playback and PsychHID for response handling via the Psychophysics Toolbox v3 library. The sound card was set to exclusive mode and driven via the Windows Session API (WASAPI) to minimize latency and jitter, and to prevent system sounds from interfering during the experiment.

### Calibration

Vibrotactile calibration involved using a digital oscilloscope (TBS1052B-EDU, Tektronix, Beaverton, OR, USA) to measure the electrical input signal driving the vibrotactile actuators. The high-frequency modulation introduced by the Class-D amplifier was filtered using a Sallen-Key second-order active low-pass filter (4954 Hz cut-off). With a 32-bit floating point digital sinusoidal signal at an intensity of 0.01 root-mean-square (RMS), the actuators received a 420 mV electrical input. This digital to analog conversion factor remained unchanged across frequencies from 100 to 1000 Hz. Based on data from the device validation study^1^, this was calculated to produce an amplitude of 55 dB peak-to-peak displacement relative to 1 micron. Auditory calibration was performed by a technician following ANSI/ASA S3.7 standard protocol^48^ for insert earphones. Measurements were done for pure tones and harmonic complex tones. The total harmonic distortion was measured below 0.25% for all tests. The data from the calibration allowed to set the auditory stimuli at 70 dB SPL for the experiments. Stimuli were adjusted during piloting to produce similarly robust yet comfortable sensations in both modalities, with selected intensities reflecting the expected faster growth of perceived intensity in the tactile compared to the auditory modalitiy^49^.

### Procedure

The study followed a quasi-experimental within-subject design. The dependent variables were the discrimination thresholds measured for six timbre-related acoustic parameters: Number of harmonics in the tone, Harmonic roll-off ratio (decrease in amplitude of subsequent harmonic), Even-harmonic attenuation (decrease in amplitude of even harmonics), Attack time (time to reach maximum amplitude), Amplitude modulation (AM) depth, and AM frequency of the harmonic complex carrier tone. For each acoustic feature, the independent variable was the sensory modality in which thresholds were measured: Auditory only (A), Auditory + Vibrotactile together (A + VT), and Vibrotactile only (VT). Each modality condition was tested in a randomized order using the same stimuli. In the A condition, stimuli were presented through insert earphones only. In the A + VT condition, stimuli were presented simultaneously through insert earphones and through the Multichannel Vibrotactile Gloves worn on both hands. In the VT condition, stimuli were presented through the Multichannel Vibrotactile Gloves only while participants wore earplugs and circumaural headphones playing white noise to avoid contribution from sounds emitted by the gloves to the response.

Participants were first shown the Multichannel Vibrotactile Gloves and told that the experiment involved auditory and tactile perceptual tasks. After providing written informed consent, participants completed the socio-demographic survey, the MMHQ, and the hearing screening. Participants were then comfortably seated in front of the computer to undergo the vibrotactile sensitivity screening. They wore the Multichannel Vibrotactile Gloves on both hands, earplugs, and circumaural headphones playing masking noise. Detection thresholds were measured for 100, 250, 500, and 800 Hz sinusoidal vibrations lasting 1 s using a 16-trial two-interval forced choice method. The participants were asked to judge which of the two intervals co-occurred with a faint vibration, and to answer by clicking the appropriate button on the keyboard. Detection thresholds were measured across both hands simultaneously and participants were instructed to respond to any sensation, regardless of hand or localization.

For the main experiment, discrimination thresholds for the six acoustic parameters were measured for each sensory modality conditions in a randomized order. An ABX forced choice method was used. For each acoustic parameter, a baseline stimulus remained consistent throughout each trial, and the difference between this baseline and a contrast stimulus was increased or lowered to estimate the discrimination thresholds. At each trial, the baseline was randomly assigned to either “A” or “B”, and the contrast to the other, whereas “X” was randomly assigned either the baseline or the contrast stimulus. A graphical user interface on the computer screen displayed an “A”, “B”, and “X” button, which turned blue one after the other while playing their respective sound and/or vibration. The participants were instructed to judge which stimulus, “A” or “B”, matched the “X” stimulus. If participants were unsure or did not know, they were instructed to guess or follow their intuition. This was repeated for 40 trials using the Bayesian adaptative staircase algorithm QUEST^50^ to estimate the threshold. The algorithm was given informative priors based on data obtained from 12 pilot participants prior to this study. After each response, the model updated its probabilistic estimation of the discrimination thresholds based on all previous responses and calculated which stimuli to present next. The QUEST algorithm was used for its ability to efficiently converge to accurate thresholds by maximizing the information gained on each trial. After the first 20 trials, the algorithm was allowed to do an early stopping if the estimated threshold converged to a stable value with a high confidence level. This is coherent with other protocols using the QUEST algorithm^51^ and with the literature showing that QUEST threshold estimates rapidly converges during the first 30–40 trials^52^, and that accurate thresholds can be obtained with as few as 18 trials when informative priors are set^53^.

### Stimuli

**Fig. 2:**
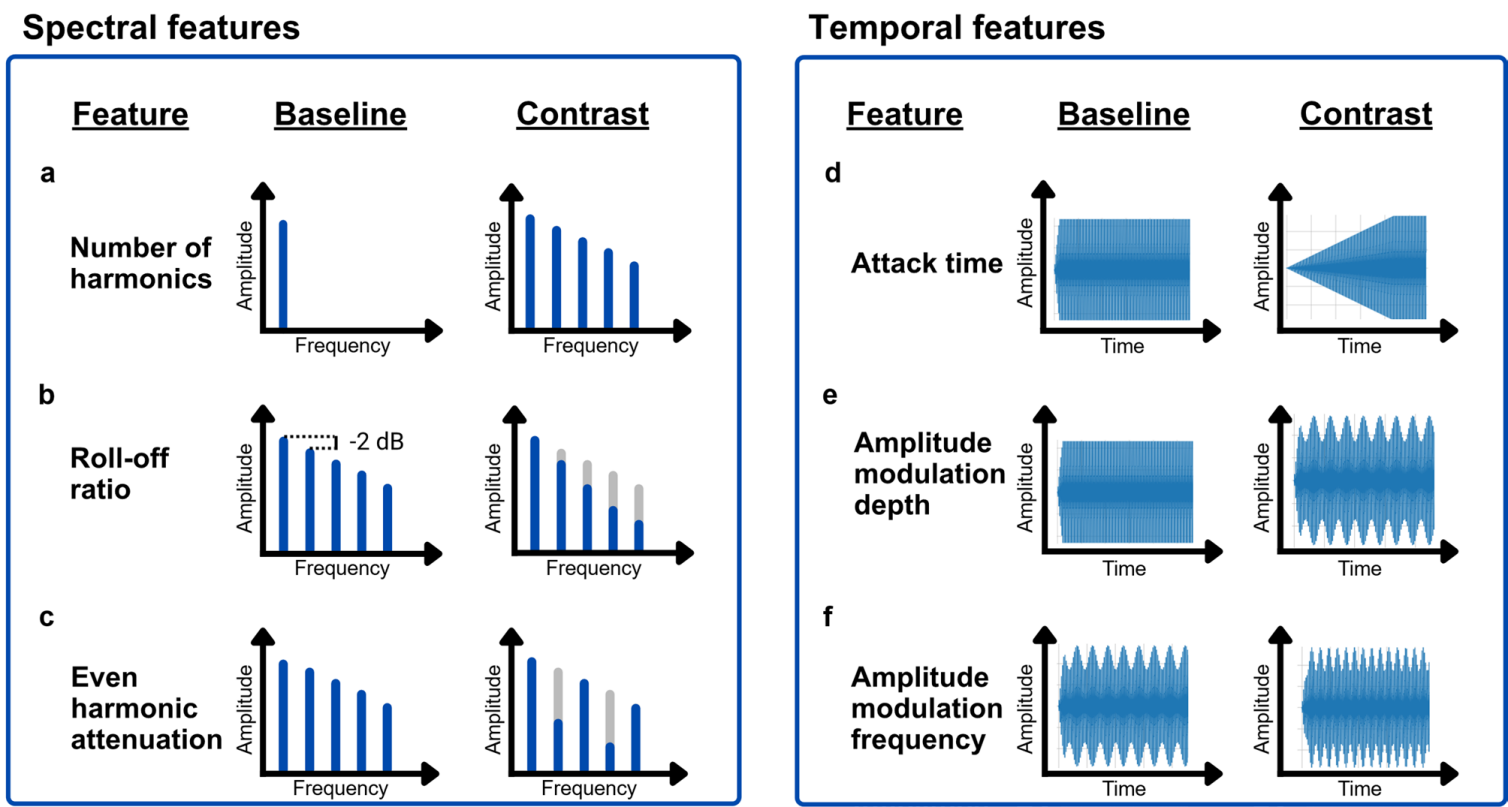
Visual representation of baseline and possible contrast stimuli for each of the six acoustic features.

Stimuli were generated by additive synthesis to vary the spectral and temporal parameters in a controlled manner^54^ for the following six acoustic parameters: Number of harmonics, Harmonic roll-off ratio, Even-harmonic attenuation, Attack time, AM depth, and AM frequency (Fig. 2). The first three concern the spectral shape of the sound, while the latter three concern its temporal envelope^9,55^.

The stimuli always had a fundamental frequency of 130 Hz, which was chosen to minimize the contribution of mechanoreceptors involved in lower-frequency perception while also maximizing the number of harmonics that could be present in the frequency response range of Pacinian corpuscles^19^. The main mechanoreceptors involved in lower frequency perception, Meissner corpuscles, are said to operate in the approximate range of 10 to 80 Hz. However, both Meissner corpuscles and Pacinian corpuscles can overlap for supra-threshold stimuli, with Meissner corpuscles only significantly dropping off in sensitivity above 100 Hz and Pacinian corpuscles reaching peak sensitivity around 250 Hz^13^. In most conditions, stimuli had 8 harmonics in addition to the 130 Hz fundamental frequency for a total of 9 sinusoids, each with a starting phase of π. This means that in theory the last two harmonics (1040 Hz and 1170 Hz) fall outside the frequency response range of Pacinian corpuscles. However, amplitude modulations of these higher frequencies can still be perceived very efficiently^56^, meaning that they could be useful to perceive changes in the temporal envelope.

Stimuli lasted 1.5 s with an inter-stimulus interval of 0.5 s and had their digital signal normalized to 0.01 RMS to reduce prominent intensity differences^57^. No correction for detection threshold were applied for the vibrotactile stimuli, as vibrotactile equal loudness contours flatten out as sensation level increases^58^ and to preserve equivalent signals across modalities and subjects. All baseline stimuli had a linear attack time of 40 msec to avoid onset artefacts from abrupt signal changes. For number of harmonics, the baseline stimulus consisted of a pure tone. For the other features, the baseline stimulus consisted of a harmonic complex tone containing the fundamental and 8 additional harmonics with a roll-off ratio of 2 dB/harmonic (Fig. 2b).

#### Number of harmonics

The contrast stimuli contained between 1 and 8 additional harmonics with a roll-off ratio of 2 dB/harmonic (Fig. 2a), meaning that each subsequent harmonic was 2 dB lower in intensity than the previous. For this parameter, the independent variable could have integer values from 1 to 8. Adding harmonics provides more energy at higher frequencies in the spectrum, thus increasing the amplitude-weighted mean frequency, simply referred to as spectral centroid (or spectral centre of gravity/mass). Spectral centroid is highly correlated with the perceptual brightness of sounds, which is one of the main dimensions of auditory timbre^59,60^.

#### Roll-off ratio

The contrast stimuli could have their roll-off values increased by 0.01 to 6 dB/harmonic in 64 steps on a logarithmic scale (Fig. 2b). Roll-off is also called harmonic amplitude decay or slope of the spectral envelope^61,62^. Higher roll-off values mean that less energy is contained in the higher frequency harmonics, thus lowering the spectral centroid^60^. This acoustic feature is thus also related to the timbre dimension of brightness, with higher roll-off ratios being associated with lower sound brightness.

#### Even-harmonic Attenuation

The contrast stimuli had their even harmonics (260, 520, 780, and 1040 Hz) attenuated by values varying between 0.1 and 25 dB in 64 steps on a logarithmic scale (Fig. 2c). This changes the odd-to-even harmonic energy ratio and increases the harmonic spectral deviation by introducing jaggedness in the spectral shape^60,63^, which is related to the timbre dimension of hollowness often associated with instruments such as the clarinet^64^.

#### Attack time

The contrast stimuli could have their attack time increased by 10 to 800 msec in 64 steps on a logarithmic scale (Fig. 2d). Attack time determines how fast the stimulus goes from an amplitude of 0 to its maximum amplitude. It is related to the timbral attribute of impulsivity^65^ and is one of the most important temporal dimensions of timbre for musical instrument identification^9,66^.

#### Amplitude modulation depth

The contrast stimuli could have 10 Hz amplitude modulations with depth values between 1 and 100% in 64 steps on a logarithmic scale (Fig. 2e). AM depth impacts the perceptual timbre attributes of fluctuation (or tremolo) and roughness, which become more salient with increasing AM depth^67,68^.

#### Amplitude modulation frequency

The same baseline stimulus was used but was additionally amplitude modulated at a frequency of 10 Hz with 25% depth. The contrast stimuli could have their AM frequency increased by 0.01 to 8 Hz in 64 steps on a logarithmic scale (Fig. 2f). AM frequency impacts whether a sound is more perceived as fluctuating or as rough. In hearing, fluctuation (or tremolo) is dominant at lower AM frequencies, while roughness is dominant at higher AM frequencies above 20 Hz^24^.

### Statistical analysis

Since the acoustic features relate to distinct perceptual dimensions yielding heterogeneous outcome variables (e.g., seconds, dB, Hz), each feature was analyzed separately using a linear mixed model (LMM) to ensure accurate estimation of effects specific to each feature’s scale and distribution. Models were fitted using the *glmmTMB* package (v1.1.10) in R (v4.4.0). LMMs were chosen to handle the lack of independence between repeated measurements within participants and for their flexibility in modeling heterogeneous variances across conditions^69^. Each model included Discrimination Thresholds as the response variable, Condition as the fixed effect, and Participant as random intercepts. The main predictor Condition (categorical: A, A + VT, VT) was dummy coded with condition A as reference. This coding scheme was selected since it matched the main research question of whether A + VT and VT thresholds are different from the baseline auditory thresholds. No random effect variance-covariance structure was specified, as the design matrix reduces to a single variance term for models with a single random effect. To account for potentially large differences in variances across different modality conditions, we specified a heterogeneous variance structure^70^ through a dispersion model by setting *dispformula = ~ Condition*^71^. This approach is functionally equivalent to a heterogenous diagonal residual matrix, but uses a log link to model estimates of the residual variance at each level of Condition, which can then be tested for significance using standard *Z* statistics.

Model significance was assessed using a Chi-square likelihood ratio test to compare models with and without the fixed effect. These models were fitted using Maximum Likelihood (ML) to allow model comparison. Further analysis was conducted on models fitted with Restricted Maximum Likelihood (REML). Model diagnostics were conducted using the *DHARMa* package (v0.4.7), which implements robust diagnostics for mixed effect models based on standardized simulated (*n* = 1000) residuals, and with the *performance* package (v0.12.4). This involved visual inspection of fixed and random effects QQ plots, within-group boxplots of residuals, and posterior predictive check, as well as computation of the Kolmogorov-Smirnov test for normality, Levene’s Test for homogeneity of variance, and a bootstrapped outlier test at both margins (nBoot = 1000). The significance for fixed effect and variance coefficients was given by a Wald *z*-test. Pseudo *R*^*2*^ values were computed using the sum of the squared residuals normalized by the total variance in the outcome variable. Details of model fitting, diagnosis, and evaluation are presented according to currently established guidelines for reporting LMMs in psychological sciences^72^ in Supplementary Note 1. Post-hoc *t*-tests with Holm correction for multiple comparisons were conducted on the estimated marginal means retrieved using the *emmeans* package (v1.10.5) and *d*_*z*_ effect sizes were obtained using the *effectsize* package (v0.8.9).

## Results

To assess the inherent ability of the tactile modality to encode spectral and temporal acoustic features typically associated with the auditory perception of timbre, discrimination thresholds for six acoustic features were compared across auditory, tactile, and multimodal auditory + vibrotactile stimulation using LMMs (Table 2).

**Table 2:** Model parameters.

Roll-off ratio											
Fixed effects	Coef	SE	Z	*p*	Residuals	Var	SD	Coef	SE	Z	*p*
*Intercept* (A)	0.41	0.03	13.77	< 0.001	*Intercept* (A)	0.02	0.14	−1.97	0.17	−11.41	< 0.001
Condition A + VT	0.03	0.03	0.93	0.35	Condition A + VT	0.01	0.12	−0.18	0.25	−0.71	0.48
Condition VT	1.78	0.07	25.80	< 0.001	Condition VT	0.13	0.36	0.95	0.23	4.11	< 0.001
					Participant	0.01	0.09	−	−	−	−

**Even-harmonic attenuation**											
**Fixed effects**	**Coef**	***SE***	***Z***	***p***	**Residuals**	**Var**	***SD***	***Coef***	***SE***	***Z***	***p***
*Intercept* (A)	4.41	0.29	15.25	< 0.001	*Intercept* (A)	1.36	1.17	0.15	0.19	0.32	0.41
Condition A + VT	0.67	0.28	2.40	0.017	Condition A + VT	1.03	1.02	−0.13	0.31	−0.43	0.66
Condition VT	9.72	0.74	13.22	< 0.001	Condition VT	15.83	3.92	1.21	0.23	5.25	< 0.001
					Participant	1.23	1.11	−	−	−	−

**Attack time**											
**Fixed effects**	**Coef**	***SE***	***Z***	***p***	**Residuals**	**Var**	***SD***	***Coef***	***SE***	***Z***	***p***
*Intercept* (A)	0.22	0.03	7.73	< 0.001	*Intercept* (A)	0.01	0.10	−2.26	0.14	*−*16.16	< 0.001
Condition A + VT	−0.05	0.02	−2.70	0.007	Condition A + VT	< 0.001	0.03	*−*1.30	0.91	*−*1.42	0.16
Condition VT	0.03	0.03	1.34	0.18	Condition VT	0.01	0.10	*−*0.05	0.20	*−*0.22	0.82
					Participant	0.01	0.12	−	−	−	−

**AM depth**											
**Fixed effects**	**Coef**	***SE***	***Z***	***p***	**Residuals**	**Var**	***SD***	***Coef***	***SE***	***Z***	***p***
*Intercept* (A)	0.09	0.01	16.32	< 0.001	*Intercept* (A)	< 0.001	0.02	−3.78	0.17	−22.58	< 0.001
Condition A + VT	−0.02	0.01	−3.39	< 0.001	Condition A + VT	< 0.001	0.02	−0.20	0.23	−0.72	0.47
Condition VT	−0.01	0.01	−1.94	0.052	Condition VT	< 0.001	0.02	0.08	0.25	0.33	0.74
					Participant	< 0.001	0.02	−	−	−	−

**AM frequency**											
**Fixed effects**	**Coef**	***SE***	***Z***	***p***	**Residuals**	**Var**	***SD***	***Coef***	***SE***	***Z***	***p***
*Intercept* (A)	2.16	0.20	10.61	< 0.001	*Intercept* (A)	0.61	0.78	−0.24	0.17	−1.46	0.14
Condition A + VT	−0.05	0.16	−0.32	0.75	Condition A + VT	0.18	0.42	−0.63	0.44	−1.43	0.15
Condition VT	2.66	0.23	11.40	< 0.001	Condition VT	1.08	1.04	0.28	0.22	1.26	0.21
					Participant	0.67	0.82	−	−	−	−

### Number of harmonics

**Fig. 3:**
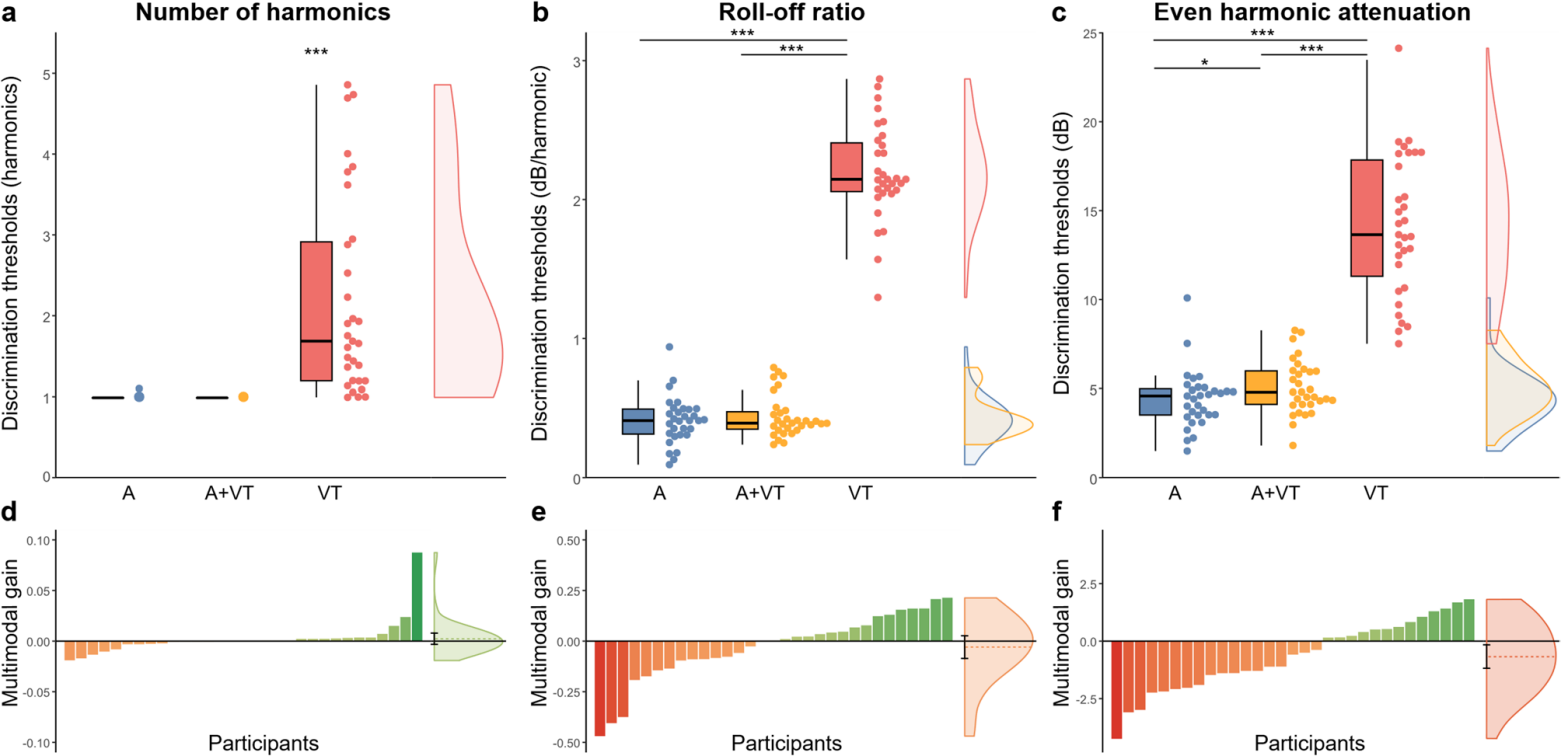
Discrimination thresholds and multimodal gain for spectral acoustic features. The top plots (a-c) show the discrimination thresholds as boxplots, individual datapoints, and distributions for auditory (A) in blue, auditory + vibrotactile (A + VT) in yellow, and vibrotactile (VT) in red stimulation conditions. Lower thresholds indicate better discrimination performance. The bottom plots (d-f) show each participant’s multimodal gain, calculated as their auditory thresholds minus their auditory + vibrotactile thresholds (A minus A + VT), in ascending order. Negative values (in red) represent a lower performance in the multimodal condition, while positive values (in green) represent better multimodal performance. The distributions on the right show the mean multimodal gain as the dotted line, with the error bar representing the 95% CI. *p <.05. **p <.01. ***p <.001.

Number of harmonics discrimination thresholds are plotted in Fig. 3a. The model for number of harmonics was judged inadequate for inference based on its diagnosis and evaluation (see Supplementary Note 1), which can be explained by a strong floor effect for A and A + VT thresholds, meaning that virtually all participants could discriminate the smallest possible contrast in these conditions (*M* ≈ 1, *Mdn* ≈ 1, SD ≈ 0.01). Future studies could alleviate this floor effect by using a harmonic complex baseline condition (e.g., starting from a baseline stimulus with 4 harmonics) or gradually fading in higher harmonics. Thresholds were higher than the minimum contrast only in the VT condition (*M* = 2.2, *Mdn* = 1.68, *SD* = 1.24). However, VT thresholds did not follow a normal distribution (*D* = 0.84, *p* <.001). As such, significance was assessed using a one sample Wilcoxon signed-rank test which revealed that the VT median was significantly different from the theoretical median of 1, *Z* = − 5.59, *p* <.001, 95% CI [1.52, 2.69], with a large effect size (*r* =.85). Overall, these results suggest that all participants were reliably able to discriminate pure tones from complex tones with one additional harmonic in the A and A + VT stimulation condition (Fig. 3d). For the VT stimulation, some participants were also able to reliably discriminate pure tones from complex tones with one additional harmonic, however most participants tended to require more harmonics in the complex tones to discriminate it.

### Roll-off ratio

Roll-off ratio discrimination thresholds are plotted in Fig. 3b. The model revealed a statistically significant effect of the VT condition relative to the baseline A condition, but no statistically significant difference was observed between the A + VT and A conditions (Table 2). Post-hoc comparisons indicated that discrimination thresholds (dB/harmonic) were significantly higher in the VT condition (*M* = 2.20, *Mdn* = 2.15, *SD* = 0.53) compared to the A condition (*M* = 0.41, *Mdn* = 0.41, *SD* = 0.17), *t*(86) = 25.80, *p* <.001, with a very large effect size (*d*_*z*_ = 4.19, 95% CI [3.09, 5.28]), and compared to the A + VT condition (*M* = 0.44, *Mdn* = 0.39, *SD* = 0.15 dB), *t*(86) = 25.87, *p* <.001, with a very large effect size (*d*_*z*_ = 4.37, 95% CI [3.23, 5.51]). However, thresholds were not significantly different between the A + VT and A conditions, *t*(86) = − 0.95, *p* =.355 (*d*_*z*_ = 0.17, 95% CI [− 0.17, 0.52]). These results suggest that vibrotactile roll-off ratio discrimination thresholds are much higher than auditory and auditory + vibrotactile discrimination thresholds, and that multimodal presentation did not positively or negatively impact discrimination thresholds (Fig. 3e).

### Even-harmonic attenuation

Even-harmonic attenuation discrimination thresholds are plotted in Fig. 3c. The model revealed statistically significant effects of the A + VT and VT conditions relative to the baseline A condition (Table 2). Post-hoc comparisons indicated that discrimination thresholds (dB) were significantly higher in the A + VT condition (*M* = 5.08, *Mdn* = 4.80, *SD* = 1.51) compared to the A condition (*M* = 4.41, *Mdn* = 4.59, *SD* = 1.62), *t*(86) = 2.38, *p* =.019, with a small to medium effect size (*d*_*z*_ = 0.42, 95% CI [0.07, 0.78]). Thresholds were also significantly higher in the VT condition (*M* = 14.13, *Mdn* = 13.65, *SD* = 3.98), compared to the A condition, *t*(86) = 13.22, *p* <.001, with a very large effect size (*d*_*z*_ = 2.29, 95% CI [1.62, 2.95]), and compared to the A + VT condition, *t*(86) = 12.44, *p* <.001, with a very large effect size (*d*_*z*_ = 2.16, 95% CI [1.52, 2.79]). These results suggest that vibrotactile even-harmonic attenuation discrimination thresholds are much higher than auditory and auditory + vibrotactile discrimination thresholds, and that multimodal presentation negatively impacted discrimination thresholds (Fig. 3f).

### Attack time

**Fig. 4:**
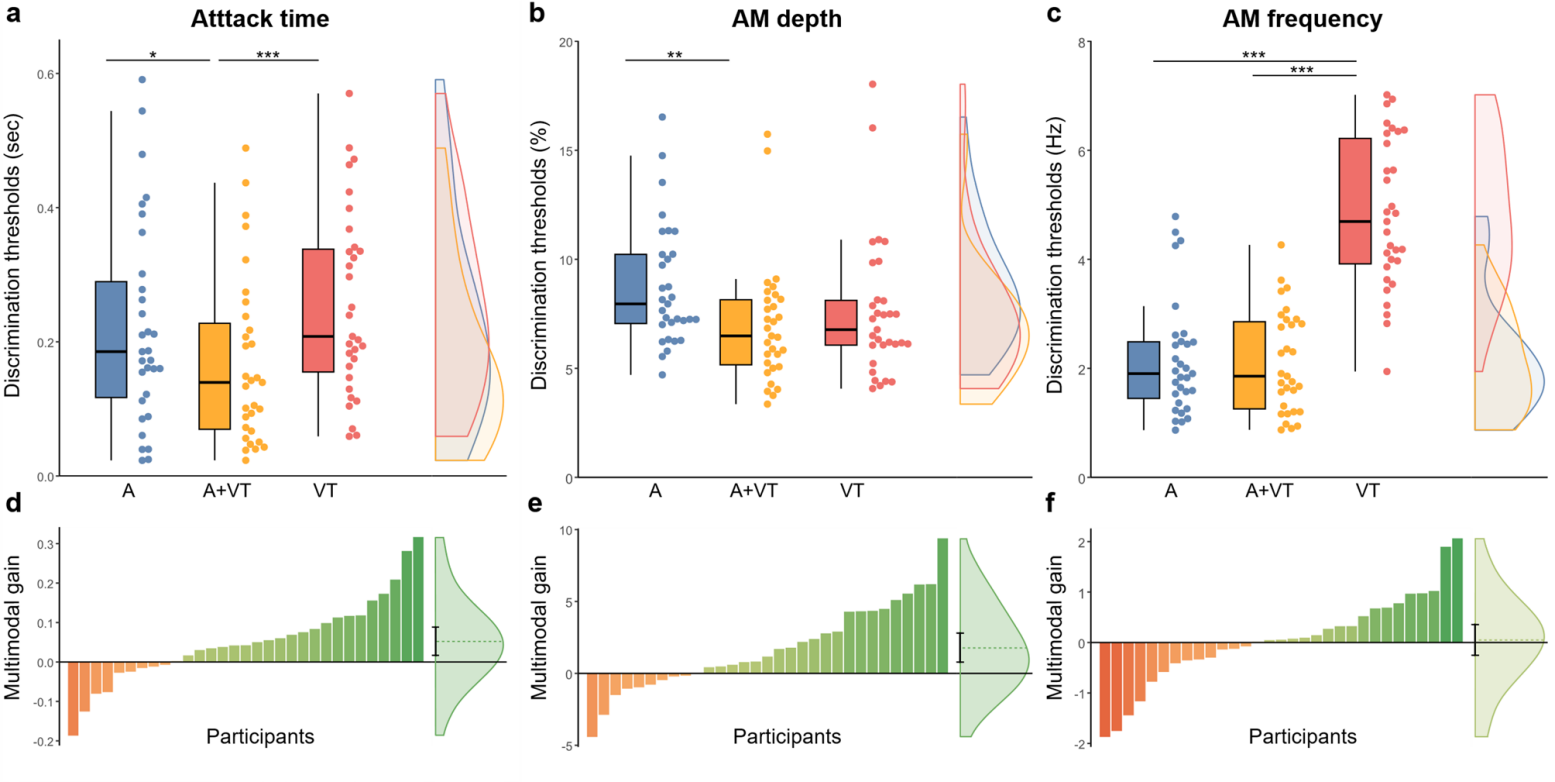
Discrimination thresholds and multimodal gain for temporal acoustic features. The top plots (**a-c**) show the discrimination thresholds as boxplots, individual datapoints, and distributions for auditory (A) in blue, auditory + vibrotactile (A + VT) in yellow, and vibrotactile (VT) in red stimulation conditions. Lower thresholds indicate better discrimination performance. The bottom plots (**d-f**) show each participant’s multimodal gain, calculated as their auditory thresholds minus their auditory + vibrotactile thresholds (A minus A + VT), in ascending order. Negative values (in red) represent a lower performance in the multimodal condition, while positive values (in green) represent better multimodal performance. The distributions on the right show the mean multimodal gain as the dotted line, with the error bar representing the 95% CI. *p <.05. **p <.01. ***p <.001.

Attack time discrimination thresholds are plotted in Fig. 4a. The model revealed a statistically significant effect of the A + VT condition relative to the baseline A condition, but no statistically significant difference was observed between the VT and A conditions (Table 2). Post-hoc comparisons indicated that discrimination thresholds (in seconds) were significantly lower in the A + VT condition (*M* = 0.17, *Mdn* = 0.14, *SD* = 0.13) compared to the A condition (*M* = 0.19, *Mdn* = 0.22, *SD* = 0.15), *t*(86) = − 2.70, *p* =.017, with a small to medium effect size (*d*_*z*_ = − 0.48, 95% CI [− 0.84, − 0.12]), and compared to the VT condition (*M* = 0.26, *Mdn* = 0.21, *SD* = 0.14), *t*(86) = − 4.68, *p* <.001, with a large effect size (*d*_*z*_ = − 0.82, 95% CI [− 1.22, − 0.42]). However, no significant difference was observed between the VT and A conditions, *t*(86) = − 1.34, *p* =.183 (*d*_*z*_ = 0.21, 95% CI [− 0.14, 0.56]). These results suggest that vibrotactile and auditory attack time discrimination thresholds are similar, and that multimodal presentation positively impacted discrimination thresholds (Fig. 4d).

### Amplitude modulation depth

AM depth discrimination thresholds are plotted in Fig. 4b. The model revealed a statistically significant effect of the A + VT condition relative to the baseline A condition, but no statistically significant difference was observed between the VT and A conditions (Table 2). Post-hoc comparisons indicated that discrimination thresholds (percent) were significantly lower in the A + VT condition (*M* = 6.98, *Mdn* = 6.49, *SD* = 2.78) compared to the A condition (*M* = 8.77, *Mdn* = 7.96, *SD* = 2.81), *t*(86) = − 3.39, *p* =.003, with a medium effect size (*d*_*z*_ = − 0.58, 95% CI [− 0.96, − 0.21]). However, no significant difference was observed between the VT (*M* = 7.60, *Mdn* = 6.77, *SD* = 3.20) and A conditions, *t*(86) = − 1.94, *p* =.112 (*d*_*z*_ = − 0.32, 95% CI [− 0.67, 0.03]), or between the A + VT and VT conditions, *t*(86) = − 1.11, *p* =.270 (*d*_*z*_ = − 0.21, 95% CI [− 0.55, 0.14]. These results suggest that vibrotactile and auditory AM depth discrimination thresholds are similar, and that multimodal presentation improved discrimination thresholds compared to auditory only presentation (Fig. 4e).

### Amplitude modulation frequency

AM frequency discrimination thresholds are plotted in Fig. 4c. The model revealed a statistically significant effect of the VT condition relative to the baseline A condition, but no statistically significant difference was observed between the VT and A conditions (Table 2). Post-hoc comparisons indicated that discrimination thresholds (Hz) were significantly higher in the VT condition (*M* = 4.82, *Mdn* = 4.69, *SD* = 1.37) compared to the A condition (*M* = 2.16, *Mdn* = 1.90, *SD* = 1.06), *t*(86) = 11.40, *p* <.001, with a very large effect size (*d*_*z*_ = 1.93, 95% CI [1.34, 2.52]), and compared to the A + VT condition (*M* = 2.11, *Mdn* = 1.86, *SD* = 0.93), *t*(86) = 13.50, *p* <.001, with a very large effect size (*d*_*z*_ = 2.43, 95% CI [1.74, 3.13]). However, thresholds were not significantly different between the A + VT and A conditions, *t*(86) = − 0.32, *p* =.748 (*d*_*z*_ = − 0.06, 95% CI [− 0.40, 0.29]). These results suggest that vibrotactile AM frequency discrimination thresholds are much higher than auditory and auditory + vibrotactile discrimination thresholds, and that multimodal presentations did not positively or negatively impact discrimination thresholds (Fig. 4f).

### Effects of covariates

The fitted LMMs did not include covariates, aiming instead to model the total effects of stimulation condition on the discrimination thresholds. However, it is possible that these effects may be moderated by variables such as Age or Sex. To assess this question, we fitted three additional models for each acoustic feature, each containing an interaction term between Condition and one of these covariates. Since adding an interaction term increases model complexity and could lead to overfitting, we compared these interaction models to the main effect only model using Bayesian Information Criterion (BIC), which penalizes additional complexity. BIC values were interpreted following the guidelines from Raftery^73^. For AM depth and AM frequency, BIC values were within 6 to 10 points higher for the models with an interaction term, suggesting strong evidence against the inclusion of interactions. For roll-off ratio, even-harmonic attenuation, and attack time, BIC values were higher than 10 for the models with interactions, suggesting very strong evidence against the inclusion of interactions. In summary, across all acoustic features, adding an interaction term did not yield enough improvement to justify the increased complexity, suggesting that Age and Sex did not meaningfully moderate the strength or direction of the main effect in the current sample.

Although the overall stimulus intensity was equalized through RMS normalization, some intensity-related cues can still contribute to vibrotactile sensation due to known nonlinearities in vibrotactile sensitivity. To assess possible contribution of intensity-cues, exploratory correlations were conducted between vibrotactile discrimination thresholds and absolute vibrotactile detection thresholds measured during the tactile acuity screening, which exhibit the typical “U-shaped” frequency response^19^. Results showed that individual differences in frequency specific detection thresholds were not associated with discrimination performance. While this does not completely rule out some contribution of intensity cues to the responses, this suggests that discrimination thresholds are not solely driven by intensity cues.

## Discussion

The purpose of this study was to better understand how spectral and temporal acoustic features related to timbre perception are perceived via vibrotactile stimulation. Three key findings emerged from this research. First, spectral acoustic features (number of harmonics, roll-off ratio, even-harmonic attenuation) could be reliably discriminated from vibrotactile stimulation only, but at significantly higher (i.e., worse) thresholds than for auditory stimulation. Second, discrimination thresholds for temporal acoustic features (attack time, AM depth, AM frequency) were generally similar across auditory and vibrotactile stimulation conditions. Specifically, vibrotactile thresholds were not significantly different from auditory thresholds for attack time and AM depth, but were significantly higher for AM frequency. Third, a significant improvement in discrimination thresholds was observed in the multimodal auditory + vibrotactile stimulation condition compared to unimodal auditory and vibrotactile stimulation for attack time and compared to unimodal auditory stimulation for AM depth (i.e., for the acoustic features which had the most similar unimodal auditory and vibrotactile thresholds).

### Vibrotactile discrimination of spectral acoustic features

The first important finding from this study is that spectral features of complex vibrations within the frequency range of Pacinian corpuscles can be reliably discriminated, although at higher thresholds than for hearing. A prior study looking at vibrotactile discrimination on the human back found compatible results to ours, as they demonstrated that participants could discriminate between musical instruments with similar temporal features but distinct spectral structures^74^. However, this study used digitally normalized real instrument sounds and could not precisely control each acoustic parameter separately, leaving unclear which of several possible timbral acoustic features contributed to this discriminability. In the present study, we measured vibrotactile discrimination thresholds by individually modifying three spectral features.

The first two features, number of harmonics and harmonic roll-off ratio, directly impacted the spectral centroid of the stimuli, which is related to the timbre attribute of brightness in hearing^59,60^. Our results suggest that discriminating sinusoidal vibrations from di-harmonic vibrations of equal power is possible for some individuals, but that most people require more harmonics in the signal. This finding aligns with prior studies showing inaccurate discrimination between sinusoidal and di-harmonic vibrotactile stimuli^75,76^. Furthermore, Senkow et al.^35^ found that pairs of vibrations with two frequency components could also not be reliably discriminated from each other in the tactile modality. However, their data suggest that a small proportion of their participants were able to discriminate the pairs with the highest spectral contrasts, which would be consistent with the inter-individual variability observed in the present study.

The third spectral feature, even-harmonic attenuation, is related to the timbre attribute of hollowness in hearing^64,77^. Here, the baseline stimulus approached a sawtooth waveform often associated with oboe-like timbres, while increasing the even-harmonic attenuation shifted the stimuli towards a rectangular waveform typically associated with clarinet-like timbres^78^. In hearing, the perceptual shift from oboe-like to clarinet-like timbres typically occurs around − 9 to −12 dB^64^, which falls below our measured vibrotactile discrimination thresholds (−14 dB). This means that the tactile modality is not able to discriminate changes in odd to even harmonic ratios that would typically result in a shift in music instrument identification in hearing, suggesting that vibrotactile perception is highly inefficient for this feature. Furthermore, we found that multimodal stimulation yielded significantly worse thresholds for even-harmonic attenuation, suggest that tactile information can impair perception if the tactile modality can’t easily process this information.

Our general finding that complex vibrations can be discriminated based on their spectral features via the tactile modality, albeit not as well as in the auditory modality, is coherent with previous research^74,79^. Research from the Bensmaïa group^57,75,80^ also found that equal-power complex vibrations can be discriminated, with discriminability being related to spectral composition rather than waveform or intensity cues. Based on these results, they argued for a psychophysical model of Pacinian corpuscles as frequency tuned mini-channels where the perception of complex vibrations is conceptualized as a tactile analog to auditory timbre perception^75,81^. While the precise mechanisms by which this spectral information is encoded remain unclear, other studies have found similarities between vibrotactile perception of complex vibrations and auditory perception. Tummala et al.^36^ investigated how complex vibrations propagate over the whole hand and found that tissue mechanics and hand morphology produce a biomechanical filter that shares similarities with the auditory filtering of the basilar membrane in the cochlea. Spectral information may also be encoded in the temporal firing patterns of Pacinian corpuscles, which exhibit highly repeatable and millisecond precision spiking responses that are analogous to those found in the auditory periphery^34,82^. The present study adds to this literature by suggesting that the main spectral features associated with auditory timbre perception can be discriminated via vibrotactile stimulation only, although at higher thresholds than for hearing.

### Vibrotactile discrimination of temporal acoustic features

The second important finding of the present study is that the auditory and tactile modalities have a generally comparable ability to discriminate temporal acoustic features of complex signals. For attack time, one of the most important attributes of timbre^9,66^, and AM depth the average auditory and vibrotactile discrimination thresholds did not show statistically significant differences, although there was a large inter-individual variability across both conditions. However, for AM frequency, vibrotactile thresholds were significantly higher than auditory thresholds.

Few studies have compared auditory and vibrotactile discrimination of these temporal features. Studies by Formby et al.^83^ and Weisenberger et al.^84^ found that vibrotactile AM detection was generally worse compared to the auditory modality, but also reported higher thresholds than those found in the current study in both modalities. These discrepancies may be explained by differences in stimuli. Whereas Formby et al.^83^ used a sinusoidal 250 Hz sinusoidal carrier, we employed a complex carrier with a 130 Hz fundamental frequency and 8 additional harmonics. Our stimuli thus had a lower fundamental frequency and a more spectral content. Previous data from Weisenberger et al.^84^ suggests that the better thresholds in the present study cannot be solely explained by the lower fundamental, thus that the additional spectral content may play a role in improving vibrotactile AM detection. However, the discrepancy could also be attributed to difference in presentation intensity, as we used a higher intensity than previous studies and since higher intensity tend to lead to lower AM depth discrimination threshold in hearing^68^. Furthermore, compared to these previous experiments, we used multiple haptic transducers covering a larger contact area as well as higher sampling rate and digital to analog bit depth. Other studies have already noted that vibrotactile AM depth detection could match or outperform auditory AM depth detection for AM frequencies between 10 and 60 Hz using a 250 Hz carrier^83,84^. The current study further suggests that carrier frequency, spectral content, and intensity also play an important role in the relative performance of auditory and vibrotactile AM depth detection and that, for some combination of those parameters, the tactile modality can outperform the auditory modality. Thus, more psychophysical investigations of modulation depth as a function of these parameters are needed to understand which combination of parameters could be used to maximize the performance of SSDs. This is particularly relevant given that existing signal processing strategies for auditory-to-vibrotactile SSDs often involve mapping or translating a given acoustic feature (e.g., the amplitude envelope of a high-frequency band beyond the vibrotactile frequency range) to amplitude modulations of lower frequency vibrations^39,40^.

For AM frequency discrimination, we found that vibrotactile thresholds were significantly worse than auditory thresholds. This result is coherent with previous results showing that vibrotactile thresholds are worse than auditory thresholds at 5 and 10 Hz baseline AM frequency using a 250 Hz carrier^83^. Thus, our results suggest that vibrotactile AM frequency discrimination remains worse than auditory AM frequency discrimination despite using a lower frequency carrier with greater spectral content. However, Formby et al.^83^ also highlighted that vibrotactile AM frequency discrimination is highly dependent on the baseline AM frequency, generally worsening with increased frequency and with a significant drop in performance at 80 Hz. Thus, it is currently unclear if the frequency content of the carrier stimulus impacts AM frequency discrimination at higher AM frequencies where vibrotactile performance is lower. For sinusoidal vibrations, the large drop in performance at higher AM frequencies possibly suggests a shift where the vibrations become perceived as complex waves with two partials rather than amplitude modulated sinusoids, leading authors to suggest that 60 Hz to 80 Hz AM frequency may be the upper limit for SSDs to adequately convey AM frequency information^85^. However, it is possible that using complex carriers which already contain multiple partials could either expand or restrict the range at which AM frequency information may be conveyed via vibrotactile stimulation. Gaining knowledge on how complex carriers impact vibrotactile AM frequency discrimination has implications for developing signal processing or feature mapping strategies for SSDs. For instance, to compensate for the lower performance of the tactile modality to detect change in AM frequency, these devices may need to expand a lower range of auditory modulation frequencies (e.g., 0–20 Hz) to a broader range of vibrotactile modulation frequencies (e.g., 0–60 Hz). The limitations in vibrotactile AM frequency discrimination should also be considered when mapping other acoustic features (e.g., spectral features) to amplitude modulation of lower frequency carriers.

### Auditory-vibrotactile interactions

The third important finding of the present study is that combined auditory + vibrotactile thresholds were significantly lower than auditory thresholds for attack time and AM depth, indicating that adding vibrotactile information can improve performance for these temporal features. Perceptual integration of auditory and vibrotactile stimulation has previously been reported for low-level perceptual processes such as intensity detection thresholds^37,38^ and for high-level perceptual processes such as speech intelligibility^39^. The present study thus sheds light on which mid-level perceptual attributes may be integrated to yield these higher-level improvements in perception. The result suggesting that multimodal presentation can lower discrimination thresholds for both attack time and AM depth is also coherent with the findings from Verma et al.^65^who investigated the multimodal perception of these two features using a multidimensional scaling approach. They observed that vibrotactile attack time and AM depth contributed to the perception of their respective timbre attributes of impulsivity and roughness/fluctuation, suggesting crossmodal processing between the auditory and vibrotactile modalities for timbre perception. According to the principle of inverse effectiveness^86^, multisensory integration is strongest when the unimodal components are weak^87^. Although its relevance to behavioral measurements remains debated^39^, our findings align with the idea that integration is facilitated when perceptual salience is balanced across modalities. For example, features such as attack time and AM depth showed improved discrimination in the audiotactile condition, suggesting that both modalities contributed meaningful cues. In contrast, when a feature was clearly more salient in one modality (typically auditory), no multisensory benefit was observed, and in some cases the addition of tactile input may have diverted attention from the more informative auditory signal. These results suggest that perceptual salience matching across modalities may be necessary to elicit integration patterns consistent with inverse effectiveness or probability summation. This has implications for experimental designs and development of SSDs, where careful selection of acoustic features, signal processing, and feature-mapping strategies may be necessary to balance salience of sensory cues across modalities and promote integration^39,88^.

### Limitations and further research

In the present study, we only assessed discrimination for complex tones with a 130 Hz fundamental frequency. This was chosen to predominantly stimulate Pacinian corpuscles afferents while allowing multiple harmonics to be present in their frequency response range. However, the effect of the fundamental frequency on complex vibration discrimination remains unexplored. Furthermore, recent findings suggest that the fundamental or lowest frequency component may play a more salient role in discriminating complex vibrations than the higher frequency components^89^. As such, vibrotactile perception may behave differently in the case of complex stimuli with missing fundamentals. The results of this study may not be entirely generalizable to other vibrotactile technologies, as each employs different types of actuators with their own frequency-dependent behaviours. Moreover, unlike auditory stimulation systems, there is currently no universally accepted standard for calibrating vibrotactile equipment, largely due to the diversity of actuator technologies and the complex interactions between the device and the skin. These factors may complicate reproducibility across different setups. However, the actuators used in this study were characterized in detail in our previous work^1^, and were selected for their affordability, commercial availability, and ease of integration in real-world applications where precise calibration remains technically challenging.

Controlling the intensity of vibrotactile stimuli is essential to ensure accurate results^57^. In this study, we normalized waveform RMS power, as Pacinian channels are thought to integrate stimulus energy over time^21,57^. While theoretically grounded and straightforward, this approach does not guarantee frequency specific equivalence in perceived intensity due to the frequency dependent nature of Pacinian corpuscles mediated vibrotactile sensitivity^19^. Some studies have attempted to equalize the contribution of frequency component by applying correction factors based on individual detection threshold^57,80^. However, this method assumes uniform growth in perceived intensity across frequencies, which does not appear to hold for higher intensity stimuli as, like in hearing, equal loudness contours flatten out at higher intensities^58^. To avoid such issues, some auditory timbre tests ask participants to subjectively match the perceived intensity across stimuli that vary in spectral content as a calibration step^90^. However, the precision of such calibration procedures in the tactile domain remains largely unknown. Another possibility would be to introduce some intensity jitter, however while this reduces the risk of intensity cues, it can also bias the response by making it harder to attend to subtle spectral cues. Overall, the effect of frequency content on perceived intensity at higher sensation levels is not well understood. Exploratory analyses found no association between individual differences in frequency specific detection thresholds and individual differences in VT discrimination thresholds. While this strengthened our results, suggesting that variations in individual sensitivity profiles did not drive task performance, it does not rule out the possibility that subtle intensity cues influenced discrimination performance. In summary, the present approach to intensity normalization balances simplicity, interpretability and theoretical validity, but the contribution of global intensity cues to spectral discrimination abilities should be further explored in future studies and validated intensity normalization procedure should be established.

Previous studies have also shown that vibrotactile perception is highly susceptible to past sensory experiences, such as multimodal training in musicians^18^ or auditory deprivation in individuals with hearing loss^91,92^. Since auditory-to-vibrotactile SSDs have many applications for both musicians and people with hearing loss^7^, it is highly relevant to also assess if there are significant perceptual differences in the complex waveform discrimination and multimodal perception for those populations. Such perceptual differences would indicate the involvement of brain plasticity and suggest that training could play a role in maximizing the effectiveness of auditory-to-vibrotactile assistive technologies. Some studies have looked at the effect of training for multimodal auditory + vibrotactile speech in noise perception^4,39,40^. Even with relatively short training protocols and a focus on higher-order auditory abilities involving speech, these studies tend to show some improvement with training. It is thus possible that training might also affect mid-level sensory processing abilities like discriminating sounds based on their timbre-related acoustic features. This would then suggest that the current results do not represent the absolute limit of vibrotactile perception, but rather a baseline that could be improved thought training. Potential benefits of vibrotactile input might also be greater when auditory information is degraded (e.g., because of noise or hearing loss), which constitute plausible use case for auditory-to-vibrotactile SSDs.

Furthermore, our results reveal substantial inter- and intra-individual variability, which is consistent with previous studies showing that, while some participants can greatly benefit from vibrotactile stimulation, others show no benefits or reduced performance in multimodal conditions^39^. We further observed that participants could perform better than average for some combinations of acoustic features and sensory modality, but below average for others. Identifying the factors underlying this variability could be instrumental in determining who is most likely to benefit from auditory-to-vibrotactile assistive technologies and in optimizing signal processing and feature mapping strategies to an individual’s specific sensory profile to provide better outcomes. Such an approach aligns with current research on precision medicine, which aims to tailor interventions based on the unique characteristics of individuals. In this approach, the performance on basic perceptual task could guide the specific configuration of an auditory-to-vibrotactile SSDs, similar to recent calls for next-generation hearing devices to account for the unique perceptual profile of the user^93^.

## Conclusion

Auditory-to-vibrotactile SSDs are actively researched as a possible way to enhance speech and music perception by conveying timbre information^65,94^. The current results show that temporal timbre information, particularly attack time and AM depth, can be efficiently perceived through vibrotactile stimulation. Notably, we observed multimodal improvements for attack time and AM depth, which were the conditions with the most similar unimodal thresholds, suggesting that multimodal improvement requires similar levels of discriminability between auditory and tactile modalities. Thus, conveying temporal features such as attack time or AM depth through SSDs may not require much signal processing as both modalities appear to have similar performance. However, for spectral features, thresholds were significantly higher for vibrotactile compared to auditory stimulation. This effect suggests that for SSDs to efficiently convey spectral features may require devising ways to code the relevant spectral features in a way that at least matches auditory discriminability. Future research on the effects of past sensory experience and training on vibrotactile and multimodal perception of timbre-related acoustic features could also provide valuable insights for improving auditory-to-vibrotactile SSDs and their adoption.

## Supplementary Information

Below is the link to the electronic supplementary material.

Supplementary Material 1

## Data Availability

The datasets generated during and/or analyzed during the current study are available from the corresponding author on reasonable request.
