# Supplementary material for: Auditory and vibrotactile interactions in perception of timbre acoustic features
Source: Sci Rep. 2025 Oct 30;15:38055. doi: 10.1038/s41598-025-21908-4 (PMC12575840; doi:10.1038/s41598-025-21908-4)
Supplement: Supplementary file 1 — Supplementary Material 1 [file 41598_2025_21908_MOESM1_ESM.pdf]

Auditory and vibrotactile interactions in perception of timbre acoustic features

Loonan Chauvette[1,2,*], Anne Sophie Grenier[1,3], Philippe Albouy[1,3], Emily Coffey[4,5,6,7], Robert Zatorre[5,6,7], and Andréanne Sharp[1,2,5]

[1]CERVO Brain Research Centre, Université Laval, Quebec City, G1E 1T2
[2]Faculty of Medicine, Université Laval, Quebec City, G1V 0A6, Canada
[3]School of Psychology, Laval University, Quebec City, G1V 0A6, Canada
[4]Concordia University, Montreal, H3G 1M8, Canada
[5]Montreal Neurological Institute, McGill University, Montreal, H3A 2B4, Canada
[6]Centre for Research on Brain, Language and Music (CRBLM), McGill University, Montreal, H3G 2A8, Canada
[7]International Laboratory for Brain, Music and Sound Research (BRAMS), Montreal, H2V 2S9, Canada
*loonan.chauvette@cervo.ulaval.ca

Notes on model fitting, diagnosis, and evaluation

For number of harmonics, model residuals diagnostics revealed a significant departure from uniformity of fixed effects, D = 0.28, p < .001, and random effects, D = 0.34, p = .001. Furthermore, the random effect variance was almost null (adjusted ICC ≈ 0), meaning that the random effect did not account for any proportion of the variance in the outcome. The model was thus judged unfit for inference and a different approach was chosen for this attribute. For all other acoustic attributes, models were judged adequate for further inference based on the diagnoses.

For roll-off ratio, model residuals diagnostics revealed uniform distribution of fixed effects, D = 0.10, p = .326, and random effects, D = 0.17, p = .327, with homogeneity of variance, $F(2, 90)$ = 0.01, p = .992, no significant effect of outliers (outlier = 1, p = 0.54), and adequate posterior predictive check. The model showed a statistically significant better fit than a null model without fixed effects, $X^2(4)$ = 272, p < .001, and provided a good predictive power, R^2 = 0.94, with a root mean square error (RMSE) of 0.22. The normalized RMSE indicated that prediction errors averaged 25% of the observed standard deviation, suggesting relatively small errors compared to the variability in the data. The random effect accounted for a relatively small proportion of the variance in the outcome (adjusted ICC = 0.13). The variance modeled in the VT condition was significantly greater than in the A condition, but the A and A+VT conditions had comparable variances (Table 2).

For even harmonic attenuation, model residuals diagnostics revealed uniform distribution of fixed effects, D = 0.07 p = .799, and random effects, D = 0.12, p = .799, with homogeneity of variance, $F(2, 90)$ = 0.46, p = .633, no significant effect of outliers (outlier = 1, p = .51), and adequate posterior predictive check. The model showed a statistically significant better fit than a null model without fixed effects, $X^2(4)$ = 176, p < .001, and provided good predictive power, R^2 = 0.80, with a RMSE of 2.32. The normalized RMSE indicated that prediction errors averaged 45% of the observed standard deviation, suggesting relatively small errors compared to the variability in the data. The random effect accounted for a relatively small proportion of the variance in the outcome (adjusted ICC = 0.17). The variance modeled in the VT condition was significantly greater than in the A condition, but the A and A+VT conditions had comparable variances (Table 2).

For attack time, model residuals diagnostics revealed uniform distribution of fixed effects, D = 0.13, p = .09, and random effects, D = 0.16, p = .40, with homogeneity of variance, $F(2, 90)$ = 0.08, p = .93, no outliers, and adequate posterior predictive check. The model showed a statistically significant better fit than a null model without fixed effects, $X^2(4)$ = 24.9, p < .001, and provided good predictive power, R^2 = 0.68, with a RMSE of 0.08. The normalized RMSE indicated that prediction errors averaged 56% of the observed standard deviation, suggesting

relatively small errors compared to the variability in the data. The random effect accounted for a large proportion of the variance in the outcome (adjusted ICC = 0.67). No significant difference in variance was observed between the levels of the fixed effect (Table 2).

For amplitude modulation depth, model residuals diagnostics revealed uniform distribution of fixed effects, $D = 0.14$, $p = .052$, and random effects, $D = 0.13$, $p = .716$, with homogeneity of variance, $F(2, 90) = 0.17$, $p = .842$, no outliers, and adequate posterior predictive check. The model showed a statistically significant better fit than a null model without fixed effects, $X^2(4) = 11.2$, $p = .024$, and provided good predictive power, $R^2 = 0.58$, with a RMSE of 0.02. The normalized RMSE indicated that prediction errors averaged 64% of the observed standard deviation, suggesting relatively small errors compared to the variability in the data. The random effect accounted for a moderate proportion of the variance in the outcome (adjusted ICC = 0.43). No significant difference in variance was observed between the levels of the fixed effect (Table 2).

For amplitude modulation frequency, model residuals diagnostics revealed uniform distribution of fixed effects, $D = 0.10$, $p = .258$, and random effects, $D = 0.16$, $p = .434$, with homogeneity of variance, $F(2, 90) = 1.58$, $p = .212$, no outliers, and adequate posterior predictive check. The model showed a statistically significant better fit than a null model without fixed effects, $X^2(4) = 109$, $p < .001$, and provided a good predictive power, $R^2 = 0.82$, with a RMSE of 0.71. The normalized RMSE indicated that prediction errors averaged 42% of the observed standard deviation, suggesting relatively small errors compared to the variability in the data. The random effect accounted for a moderate proportion of the variance in the outcome (adjusted ICC = 0.52). The variance modeled in the VT condition was significantly greater than in the A condition, but the A and A+VT conditions had comparable variances (Table 2).
